# Case Report: Mild and complete thyroid peroxidase deficiency in a family with literature review

**DOI:** 10.3389/fmed.2025.1562277

**Published:** 2025-07-01

**Authors:** Xiaobi Wu, Yuerong Yan, Hongshi Wu, Kwan Leong Woo, Muchao Wu, Li Yan, Yan Li, Jin Zhang

**Affiliations:** ^1^Department of Endocrinology and Metabolism, Dongguan Kanghua Hospital, Dongguan, China; ^2^Department of Endocrinology, Sun Yat-sen Memorial Hospital of Sun Yat-sen University, Guangzhou, China

**Keywords:** *TPO* gene mutation, mild TPO deficiency, goiter, elevated FT3/FT4 ratio, thyroglobulin

## Abstract

Mild thyroid peroxidase (TPO) deficiency is an extremely rare autosomal recessive genetic disorder, with fewer than 10 cases reported globally. This condition is often misdiagnosed as primary hypothyroidism. We report a family with mild and complete TPO deficiency due to *TPO* gene mutations, including a novel mutation (p.R584W; c.1750 T > G). The literature was reviewed to provide references for early clinical diagnosis and treatment. The proband was a 27-year-old man with a 20-year history of goiter and abnormal thyroid function. Thyroid function tests showed decreased thyroxine and free thyroxine levels, increased triiodothyronine and free triiodothyronine levels, elevated FT3/FT4 ratios, normal thyroid stimulating hormone levels, and elevated thyroglobulin levels. Ultrasound highlighted goiter with multiple nodules. Previous treatment with levothyroxine (L-T4) showed no improvement in goiter nor thyroid function, leading to discontinuation. Genetic sequencing revealed a heterozygous *TPO* gene mutation (p.R584W; c.1750 T > G), predicted to be harmful by software including REVEL, PolyPhen2, and MutationTaster (REVEL score: 0.959). The proband’s brother carried the same mutation albeit with different clinical manifestations, diagnosed as complete TPO deficiency. Moreover, the clinical characteristics and gene mutations of the nine previously reported cases of mild TPO deficiency were reviewed and summarized. Hence, this study reported a family with mild and complete TPO deficiency due to *TPO* gene mutations, and the literature was reviewed to enhance clinicians’ understanding of the disease. Gene mutations aid in diagnosis. This is the first study to report the p.R584W; c.1750 T > G gene mutation, enriching the gene pool for this rare disease. The efficacy of L-T4 treatment for mild TPO deficiency requires further observation and research.

## 1 Introduction

Thyroid peroxidase (TPO) is a key enzyme in thyroid hormone synthesis. Its deficiency can lead to varying degrees of iodide organification defect (IOD) (1). Clinically, TPO deficiency is classified into two main categories, namely complete TPO deficiency and mild TPO deficiency, with the latter being extremely rare. Mild TPO deficiency, which causes a partial IOD (PIOD), is an autosomal recessive disorder characterized by late-onset goiter, elevated free triiodothyronine (FT3)/free thyroxine (FT4) ratio, normal or slightly elevated thyroid stimulating hormone (TSH), and increased thyroglobulin (Tg) (2). Due to partial loss of TPO enzymatic activity, patients can still produce some thyroid hormones during the first few years of life. Most cases are diagnosed around the age of 10 years due to progressive goiter. To date, there have been nine reported cases of mild TPO deficiency at home and abroad. These cases are characterized by elevated FT3/FT4 ratios, with residual TPO enzymatic activity ranging from 30 to 65% (3–5). In contrast, classic TPO deficiency results in a total IOD (TIOD), manifested as severe congenital hypothyroidism (CH), elevated TSH, and reduced thyroxine (T4), often detected through newborn screening (6).

Here, we report clinical data and genetic testing results for two brothers who exhibited mild and complete TPO deficiency leading to CH due to *TPO* gene mutations. Additionally, the relevant literature was retrospectively analyzed to provide references for early clinical diagnosis and treatment.

## 2 Case description

### 2.1 Case 1

The proband was a 27-year-old man who presented to the authors’ department in 2020 with a history of goiter and abnormal thyroid function for over 20 years (starting at 5 years of age). He had been evaluated at multiple hospitals in Beijing and Shanghai for goiter, where thyroid function tests showed decreased T4 and FT4 levels, increased triiodothyronine (T3) and FT3 levels, normal TSH levels, and no Tg measurement. Thyroid ultrasound indicated goiter. The proband was diagnosed with “nodular goiter” and recommended for thyroidectomy, which his family declined. Between 5 and 20 years, his thyroid progressively enlarged. At 12 years, the proband was diagnosed with “hypothyroidism” and was administered levothyroxine (L-T4) 25 μg for treatment. After 1 month, there were no significant changes in thyroid function, and the thyroid did not shrink; hence, medication was discontinued. Annual TSH monitoring since then showed no increase; moreover, his neck swelling did not progress after 20 years of age. Over the past 10 years, the proband’s weight remained stable, and he did not experience symptoms such as fatigue, edema, heat intolerance, excessive sweating, or palpitations. His growth, development, and intelligence were normal. Physical examination revealed a height of 188 cm and weight of 75 kg. The thyroid was grade 3 enlarged, soft, and had a 4 × 2 cm palpable nodule in the left and right lobes.

Thyroid function tests (Table 1) indicated decreased T4 and FT4 levels, increased T3 and FT3 levels, elevated FT3/FT4 ratios, normal TSH levels, and Tg more than 300 ng/mL (normal range: 0–20 ng/mL). Anti-TPO and anti-Tg antibodies were normal; TSH receptor antibody (TRAb) was 0.30 U/L (normal range: 0–1.75 U/L), immunoglobulin G was 40.649 g/L (normal range: 0.03–2.01 g/L), and calcitonin was 4.0 pg./mL (normal range: 0–18.2 pg./mL). Thyroid ultrasound (Table 1) suggested diffuse goiter with multiple mixed and solid nodules in both lobes, classified as ACR TIRADS 2–3. The dimensions of the largest nodule were 47 × 43 × 32 mm.

**Table 1:** Mild TPO deficiency patient data.

Changes in thyroid function, related antibodies, and thyroglobulin in Case 1.									
Date	T4 58.1–140.6 nmol/L	FT4 11.5–22.7 pmol/L	T3 0.92–2.79 nmol/L	FT3 3.5–6.5 pmol/L	TSH 0.55–4.78 mU/L	FT3/FT4	Anti-TPO 0–60 IU/mL	Anti-TG 0–60 IU/mL	Tg ng/mL
Feb 7, 2014	27.1↓	–	3.01↑	–	2.798	–	< 28	< 20	–
Dec 15, 2020	6.6↓	3.49↓	2.79	7.37↑	2.868	2.11	< 28	< 15	–
Nov 1, 2021	12.9↓	4.12↓	4.82↑	10.77↑	2.853	2.61	< 28	< 15	–
Nov 29, 2022	12.8↓	2.89↓	4.58↑	6.45	6.21↑	2.23	< 28	< 15	> 300

Changes in thyroid ultrasound							
Date	Dimensions of the left thyroid lobe	Dimensions of the right thyroid lobe	Isthmus anteroposterior diameter	Dimensions of the largest nodule in the left lobe	Dimensions of the largest nodule in the right lobe	Dimensions of the isthmus nodule	ACR TIARDS
Dec 14, 2020	40 + *23*37 mm	40 + *24*55 mm	15 mm	24*17 mm	46*27 mm	8*8 mm	Category 2–3
Mar 31, 2022	40 + *28*42 mm	40 + *37*53 mm	16 mm	34*26*18 mm	46*43*26 mm	19*7*5 mm	Category 2–3
Nov 29, 2022	40 + *33*42 mm	40 + *41*51 mm	15 mm	40*30*23 mm	47*43*32 mm	15*8*13 mm	Category 2–3

Patient, male, 26 years old. “–” denotes missing data.

Thyroid size measurement method (superoinferior diameter * anteroposterior diameter * transverse diameter); the maximum measurable superoinferior diameter by the ultrasound probe is 40 mm.

Peripheral blood was taken from the patient for next-generation sequencing (performed by Guangzhou Jiajian Medical Science Detection Co.). Genetic analysis revealed that the patient carried a heterozygous variant of the *TPO* gene on chromosome 2, namely NM_000547.5: c.1750C > T: p.R584W (heterozygous) (Table 2).

**Table 2:** Next-generation sequencing results.

Gene name	OMIM number	HG19 location	Transcript	Nucleotide and amino acid changes	Zygosity	ACMG variant classification
*TPO*	606765	chr2: 1491745	NM_000547.5	c.1750C > T (p.R584W)	Heterozygous	Class 3—variants of uncertain significance

1. ACMG: American College of Medical Genetics and Genomics.

2. Chromosome Location: Referencing the human genome version Human GRCh37/hg19.

3. According to the ACMG guidelines (2015 edition), this variant is classified as 3—variants of uncertain significance. The existing literature suggests that TPO protein mutations may be associated with type 2A hypothyroidism, an autosomal recessive condition characterized by significantly low thyroid hormone levels and goiter after birth (PMID:28648508).

The diagnosis was as follows: 1. Mild TPO deficiency (c.1750C > T: p.R584W); 2. Multiple nodules in both thyroid lobes.

For the treatment strategy, regular thyroid function monitoring and thyroid ultrasound were recommended. If necessary, a fine-needle aspiration biopsy should be performed to determine the nature of the thyroid nodules. The patient was advised to seek genetic counseling and prenatal diagnosis when planning to have children. Thyroid function tests and mutation detection were also recommended for the family.

### 2.2 Case 2

The older brother of Case 1, aged 34 years, was diagnosed with CH accompanied by goiter at 1 year due to unstable walking and delayed growth and development. He subsequently started oral L-T4 treatment, the dosage of which was regularly adjusted based on routine thyroid function tests. At the time of the study, the patient was receiving 100 μg L-T4 daily. Physical examination showed a height of 178 cm, weight of 70 kg, and grade 3 goiter that was soft in texture, without palpable nodules. Genetic testing results were the same as those in Case 1. Case 2 had one son and one daughter, both of whom exhibited no gene mutations on genetic testing, with normal thyroid function and no goiter on ultrasound.

The diagnosis was CH (c.1750C > T: p.R584W).

For the treatment strategy, the patient was advised to continue regular oral administration of L-T4, with periodic monitoring of thyroid function and thyroid ultrasound.

Regarding family history, the parents were non-consanguineous. Previous thyroid function tests indicated normal results and no history of goiter. Nevertheless, both parents exhibited elevated levels of TPO antibodies. Genetic testing revealed that the mother had low-level mosaicism for the *TPO* gene mutation NM_000547.5: c.1750C > T: p.R584W (with a zygosity of 7.91%), whereas the father did not carry the *TPO* gene mutation.

## 3 Discussion

TPO deficiency is a group of autosomal recessive disorders (7). Based on the mutation, TPO deficiency is divided into two types: PIOD and TIOD (7). In patients with PIOD, radioactive iodine levels drop by 10–50% after a perchlorate discharge test, whereas in patients with TIOD, levels drop by >90%. In normal individuals, radioactive iodine levels drop to <10%. Complete TPO deficiency can lead to TIOD, with a CH incidence rate of 1 in 2000–4000 (8). This is mainly due to thyroid dysgenesis and thyroid hormone synthesis disorders, with TPO deficiency being the most prevalent cause (2, 3). CH exhibits typical clinical manifestations, including significantly elevated TSH and Tg levels, decreased T4 and FT4 levels, reduced T3 and FT3 levels, and associated developmental delays, making it easy to diagnose through neonatal screening (6). PIOD, induced by mild TPO deficiency, is primarily characterized by delayed-onset goiter, elevated FT3/FT4 ratios, normal or mildly elevated TSH levels, and elevated Tg levels (2). These clinical manifestations are closely related to the degree of TPO enzyme activity reduction.

Here, we report two brothers with the same *TPO* gene mutation, resulting in mild TPO deficiency in the older brother and complete TPO deficiency in the proband. The older brother presented with CH, which was easy to recognize clinically. Therefore, we focused on the proband. Owing to residual TPO activity, the proband presented in childhood with goiter and abnormal thyroid function. Specifically, thyroid function tests indicated decreased T4 and FT4 levels, elevated T3 and FT3 levels, increased FT3/FT4 ratios, normal TSH levels, elevated Tg levels, and normal thyroid-related autoantibodies. Treatment with L-T4 was not effective. To date, nine cases of PIOD with an elevated FT3/FT4 ratio as a common feature of mild TPO deficiency have been reported (five abroad and four domestically). Including this case, 10 patients’ clinical characteristics were reviewed. The median age of onset was 10 years, with four women and six men. Common features included pronounced goiter and abnormal thyroid function, indicated by decreased T4 and FT4 levels, increased T3 and FT3 levels, elevated FT3/FT4 ratios, normal or mildly elevated TSH levels (not exceeding 10 mIU/L), and significantly increased Tg levels. Meanwhile, the levels of anti-TPO, anti-Tg, and TRAb were all normal. All patients received L-T4 treatment (dose not reported), with three showing improvement in goiter, three showing no change, and one experiencing further enlargement of the thyroid. The responses in the remaining three cases were not reported. Details on thyroid function, thyroid size, response to L-T4 treatment, gene mutation sites, and nucleotide changes are summarized in Table 3. Among the cases, three siblings from Japan shared the same gene mutation site, G1687T/1808-13del. Another three cases (one from Japan, two from China) shared the same gene mutation site, C670-672del, while the remaining four patients had different gene mutations.

**Table 3:** Summary of gene results and clinical characteristics of 10 patients with mild TPO deficiency reported in literature.

Case	Mutation site		Exon	Zygosity	Origin	Goiter	L-T4 dose (ugqd)	L-T4 response	Thyroid function								Perchlorate discharge rate	Thyroid ultrasound
	Nucleotide change	Amino acid change							T4	FT4	T3	FT3	FT3/FT4	TSH	Tg	Anti-TPO anti-Tg		
Case 1 (8-year-old girl)	C670-672del	p.A224del	–	Compound Heterozygous	Mother	Yes	–	Improved	–	0.99	–	4.1	0.49↑ (0.33–0.41)	6.5↑	1057↑	Normal	36.5%↑	68 mL
Case 2 (8-year-old boy)	–	p.T527c	–	Homozygous		Yes	–	Worsened	3.5↓	–	2.2↑		T3/T4 0.075↑ (0.010–0.027)	5.9↑	250↑	Normal	–	–
Case 3 (12-year-old boy)	G1687 1808.13del	G533C A574/L575del	9	Compound Heterozygous	Father	Yes	–	–	–	11.6	–	5.5	0.47	4.0	123↑	Normal	83.7%↑	45*20 mm
Case 4 (10-year-old boy)	Same as Case 3	–	10	Same as Case 3	–	Yes	–	–	–	9.0↓	–	6.0	0.67	6.0↑	129↑	Normal	76.3%↑	50*30 mm
Case 5 (8-month-old girl)	Same as Case 3	–	10	Same as Case 3	–	Yes	–	–	–	16.8	–	7.9↑	0.47	9.2↑	577↑	–	–	32.9 mm (30.8 mm)
Case 6 (27-year-old man)	C1631C > T C1921del	p.Ala544Val p.Glu641Lys p.G641L	10 11	Heterozygous	–	Yes	50	No change	32.49↓	< 5.15↓	2.26↓	5.21	> 1	2.47	> 500↑	Normal	–	Right 39*50 mm Left 39*48 mm
Case 7 (18-year-old woman)	C1009G > A	p.Glu337Lys p.Ala544Val	8 10	Compound heterozygous	–	Yes	25	Reduced	43.59↓	8.17↓	2.15↓	4.55	0.557	3.35	> 500↑	Normal	37.3%↑	Right 47*63 mm Left 40*52 mm
Case 8 (15-year-old girl)	C.670-672del	p.Asp224del p.Arg665Gln	7 11	Heterozygous	–	Yes	25	No change	44.61↓	7.91↓	2.54↓	6.08↑	0.769	6.205↑	> 500↑	Normal	–	Right 25*37 mm Left 40*52 mm
Case 9 (5-year-old boy)	C.670-672delGAC	p.Asp224del p.Cys808Ala	7 14	Heterozygous	None	Yes	–	Reduced	74.09	10.44	3.45	7.05↑	0.675	7.706↑	> 500↑	Normal	–	Right 25*30 mm Left 24*32 mm
Case 10 (26-year-old man)	C1750C > T	p.R584W	2	Heterozygous	None	Yes	–	No change	8.2↓	3.65↓	2.79	7.37↑	2.01	2.868	> 300↑	Normal	–	Right 40*37*53 mm Left 40*26*42 mm

1. L-T4, levothyroxine; TPO, thyroid peroxidase; TSH, thyroid stimulating hormone; T3, triiodothyronine; T4, thyroxine; FT3, free triiodothyronine; FT4, free thyroxine; Tg, thyroglobulin.

2. Reference ranges for the thyroid function of Cases 1 and 2: TSH 0.5–5 mU/L, T4 6–13.8 ug/dL, FT4 0.9–1.8 pg/mL, T3 0.6–1.81 ng/mL, FT3 2.6–4.9 pg/mL. Tg < 30 ng/mL (4).

3. Reference ranges for the thyroid function of Cases 3–5: TSH 0.51–4.34 uIU/mL, FT4 10.6–21.0 pmol/L, FT3 3.1–7.5 pmol/L. Tg < 30 ng/mL (5).

4. Reference ranges for the thyroid function of Cases 6–9: TSH 0.35–4.94 uIU/mL, T4 62.67–150.84 nmol/L, FT4 9.01–19.04 pmol/L, T3 2.63–5.7 noml/L, FT3 2.63–5.7 pmol/L. Tg 3.5–77 ng/mL (3).

5. Case 10 is the case reported in this study.

6. “–” denotes missing data.

These 10 cases of mild TPO deficiency leading to PIOD revealed several clinical characteristics, including early childhood onset, goiter, elevated FT3/FT4 ratios, elevated Tg levels, and normal anti-TPO and anti-Tg levels. Four patients, including the proband, had normal TSH levels, whereas the remaining six exhibited mildly elevated TSH levels (all <10 mIU/L). This condition is often misdiagnosed as hypothyroidism due to decreased T4 and FT4 levels and is consequently treated with L-T4. However, the clinical efficacy is often limited. Notably, unlike hypothyroidism, which presents with elevated TSH levels, decreased T4 and FT4 levels, and normal or decreased T3 and FT3 levels, usually caused by autoimmune thyroiditis with positive TPO antibodies, this condition features negative TPO antibodies.

Patients with PIOD often present with significant goiter as the initial symptom; however, the degree of goiter is not directly correlated with TSH levels. Despite exhibiting normal TSH levels, proband and two cases reported by Ruijin Hospital (27-year-old man and 17-year-old girl) still had grade 3 goiters, which did not reduce in size after treatment. The extent of goiter depends not only on TSH levels but also on the duration of hyperthyrotropinemia (9). Additionally, the direct action of the mutant TPO protein contributes to the formation of goiter (4). As the body’s demand for thyroid hormone increases with growth and development, the lack of TPO protein prevents the thyroid from synthesizing sufficient thyroid hormones, thereby leading to compensatory enlargement of the thyroid. Consequently, as the age of patients with PIOD increases, and the goiter often progressively enlarges. Meanwhile, the enlargement of the thyroid itself compensates for the reduced synthesis of thyroid hormones, which is why the clinical symptoms of hypothyroidism were not obvious in the patient in this study. This suggests that the degree of TPO deficiency in this patient was relatively mild, possibly due to the small degree of structural and enzymatic activity damage in the protein. This high retention of enzymatic activity resulted in less severe impairment of thyroid function.

Another common feature of patients with IOD is abnormal thyroid function, manifested as elevated T3 and FT3 levels, decreased T4 and FT4 levels, increased FT3/FT4 ratios, and normal or mildly elevated TSH levels (all <10 mIU/L). In normal individuals, T4 is directly secreted by the thyroid. Only about 20% of T3 is secreted by the thyroid, whereas the remaining 80% is produced by the peripheral conversion of T4 by removing a single 5′ iodine atom. Narumi et al. reported that in patients with mild TPO deficiency, T3 was secreted by the thyroid, and the conversion of T4 to T3 in peripheral tissues was impaired (4). The thyroid of patients with mild Tg deficiency usually prioritizes T3 secretion, which reflects chronic overstimulation of the gland (10). Narumi et al. speculated that similar adaptive responses may occur in patients with mild TPO deficiency (4), and elevated FT3/FT4 ratios may explain why their metabolism remained normal despite low T4 levels.

Elevated Tg levels is another common feature in patients with IOD. Exons 8, 9, and 10 encode the catalytic center of the TPO protein, and TPO enzyme activity depends on correct folding, membrane insertion, and intact catalysis (11). Abnormal TPO molecules can lead to defects such as TPO’s inability to bind heme, inability to bind Tg as a substrate, and abnormal cellular localization of TPO (12). Because the denatured TPO protein cannot facilitate the binding of Tg and iodine atoms, serum Tg levels are significantly elevated in these patients.

**Figure 1:**
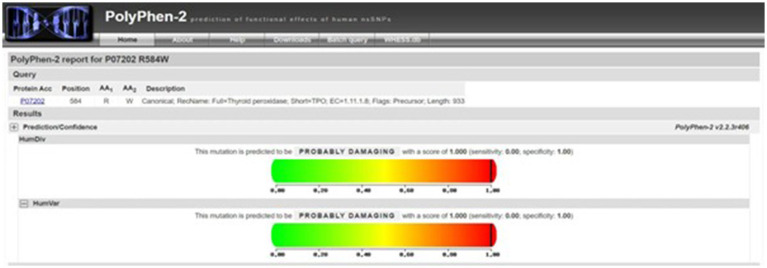
Polyphen2 prediction of mutation harmfulness: predicted as harmful mutation.

**Figure 2:**
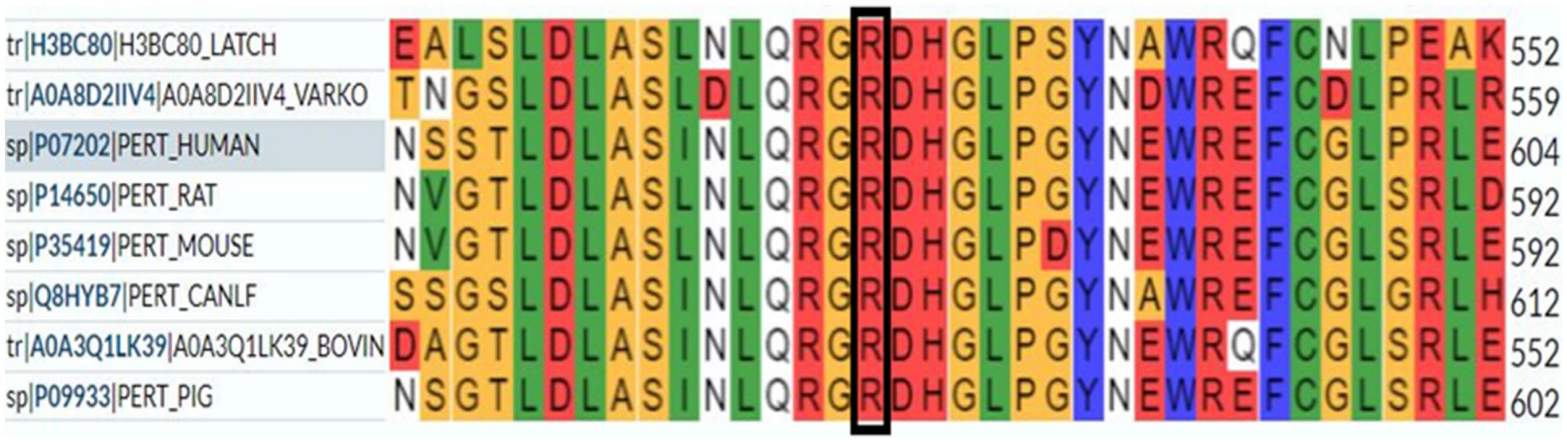
Conservation prediction indicates that the mutation site is highly conserved across different species.

**Figure 3:**
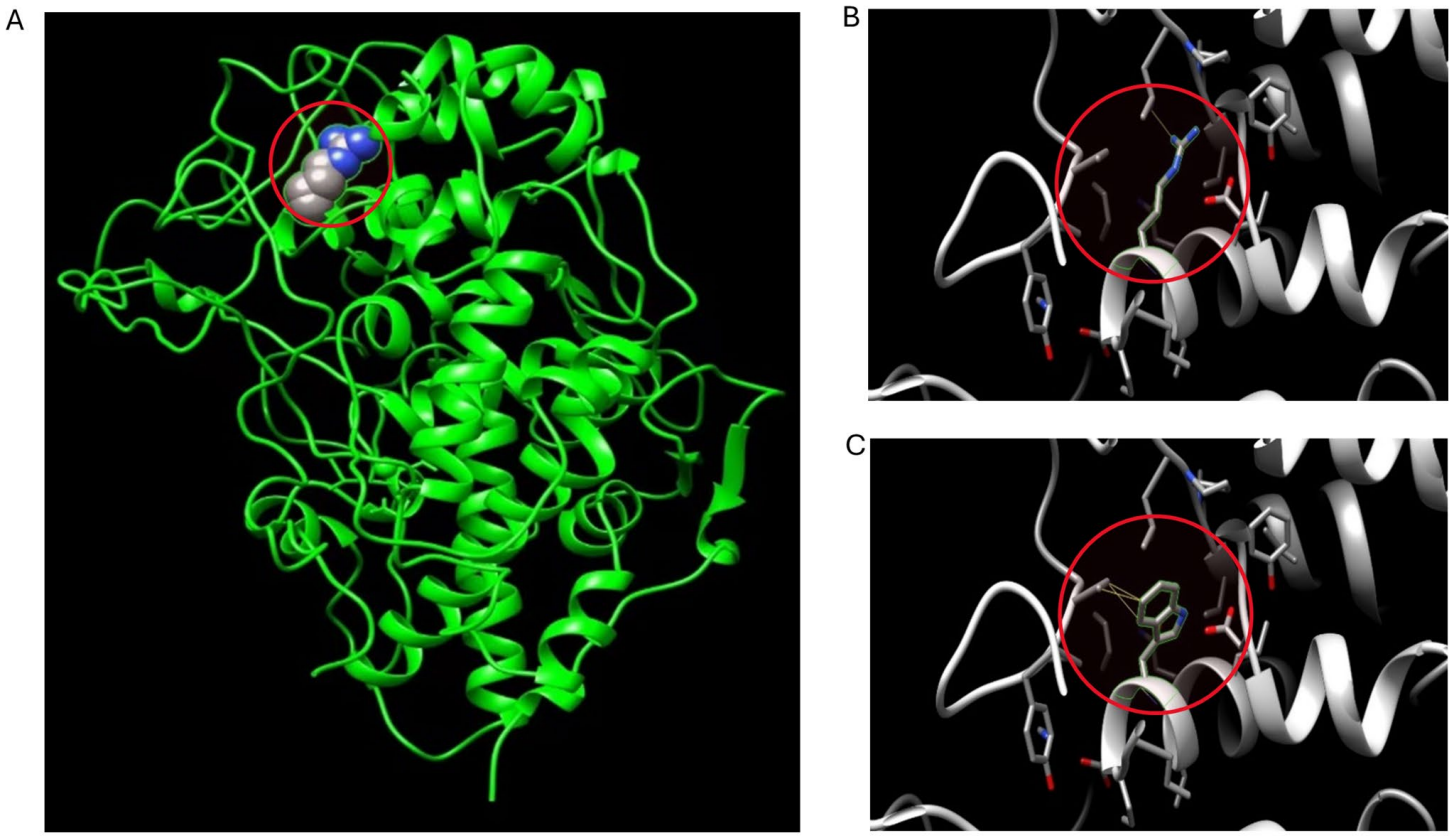
3D conformation prediction of wild-type and mutant TPO proteins. This patient’s *TPO* gene mutation results in an amino acid change at position 584 of the TPO protein (from arginine to tryptophan). **(A)** 3D conformation of the amino acid at position 584 in the mutation site (arginine) of wild-type TPO protein, highlighted in the red circle; **(B)** Micrograph of the amino acid at position 584 (arginine) in the 3D conformation of wild-type TPO protein, highlighted in the red circle; **(C)** Micrograph of the amino acid at position 584 (tryptophan) in the 3D conformation of mutant TPO protein (p.R584W), highlighted in the red circle.

According to our literature search, the *TPO* mutation (NM_000547.5: c.1750C > T: p.R584W) we observed has not been reported previously. This variant was listed in the gnomAD database as a rare mutation with an allele frequency of 0.000012. Further analysis using software predictors like REVEL, Polyphen2 (Figure 1), and MutationTaster predicted it to be a harmful variant (REVEL score: 0.959). This mutation is not listed in the ClinVar database, suggesting that it is a novel mutation. According to the ACMG guidelines (2015 edition), this mutation is classified as a variant of uncertain significance. The literature suggests that *TPO* mutations may be associated with type 2A hypothyroidism, an autosomal recessive condition characterized by significantly low thyroid hormone levels and goiter after birth (PMID:28648508). Conservation predictions indicated that the mutation site was highly conserved across different species (Figure 2), and the resultant amino acid change at position 584 in the TPO protein (from arginine to tryptophan) could impact the protein’s structure and function (Figure 3). The mutation NM_000547.5: c.1750C > T: p.R584W resulted in a nucleotide change from cytosine to thymine at position 1750 in the coding region of the *TPO* gene, leading to an amino acid substitution at position 584 from arginine to tryptophan. Furthermore, the site was highly conserved across multiple species, suggesting that the amino acid change could alter the TPO protein structure, ultimately impairing or eliminating TPO enzyme activity. Nevertheless, further validation through *in vitro* functional tests is required. The milder symptoms in Case 1 suggest that although TPO activity was partially compromised by the mutation, some functionality was retained. In contrast, Case 2, with a complete TPO mutation, presented with CH and required lifelong L-T4 therapy.

The efficacy of L-T4 in controlling goiter in patients with classic or mild TPO deficiency phenotypes is generally poor and may even exacerbate thyroid enlargement (13). In four patients with mild TPO deficiency reported by Ruijin Hospital, two patients did not show improvement in thyroid function or alleviation in goiter after L-T4 treatment, whereas the other two saw a reduction in goiter volume (3). In our study, the symptoms of the patient did not improve with L-T4 treatment, and the thyroid did not further enlarge after discontinuing the medication. Theoretically, oral L-T4 can reduce TSH levels, thereby decreasing goiter volume. However, long-term impaired thyroid hormone synthesis may lead to a relatively high set point for the TSH-thyroid hormone regulatory axis, potentially requiring lower TSH levels to suppress further goiter enlargement. Other factors related to the development of goiter may be involved in this process. For example, iodide can inhibit thyroid epithelial cell proliferation (3). Therefore, due to the lack of iodide, *TPO* mutations may increase the risk of multinodular goiter and even thyroid cancer. Consequently, patients with mild TPO deficiency may respond poorly to L-T4 treatment, necessitating surgical intervention if the patient experiences tracheal compression due to significant goiter. To determine whether mild TPO deficiency requires treatment, further clinical observations are required, given the limited number of current clinical cases.

The incidence of thyroid cancer in patients with nodular goiter is approximately 3–5% (14). Reduced TPO expression and overexpression of shorter splice variants in thyroid tissue are associated with follicular thyroid tumors (15). Patients with CH due to *TPO* gene mutations are subject to a higher risk of developing thyroid cancer than other populations (16). Nevertheless, there have been no reports of thyroid cancer in patients with mild TPO deficiency. Patients with CH and nodular goiter can develop thyroid cancer [follicular thyroid cancer reported (17, 18)]. Therefore, such patients should be assessed for the malignancy of all suspected nodules, with particular attention to the risk of developing follicular thyroid carcinoma. Since the relationship between mild TPO deficiency and thyroid cancer is not fully elucidated and due to the limited number of reported cases and the absence of thyroid cancer in the previously reported cases, long-term follow-up and regular neck ultrasounds are warranted for these patients. If necessary, fine-needle aspiration biopsy of the thyroid should be performed to confirm the presence of follicular thyroid carcinoma.

Clinically, identifying and screening high-risk individuals for mild TPO deficiency is essential. Patients with goiter and normal TPO antibodies should be carefully evaluated. Because T4 and FT4 levels are decreased, these patients are easily misdiagnosed with hypothyroidism and inappropriately treated with L-T4 replacement therapy. Clinicians should pay close attention to patients with abnormal thyroid function, with specific manifestations including elevated FT3/FT4 ratios, decreased T4 and FT4 levels, elevated T3 and FT3 levels, and normal or mildly elevated TSH levels, especially when TPO antibodies are negative. Even if TSH levels are normal, mild TPO deficiency should be considered. Additionally, Tg testing is recommended, while genetic testing and the perchlorate discharge test can help diagnose and differentiate mild TPO deficiency early. For patients with mild TPO deficiency, the decision to undergo thyroid hormone replacement therapy requires more clinical data due to the small sample size of the existing research. If there is no TSH elevation, L-T4 treatment usually provides no benefits, and follow-up is recommended. Conversely, when goiter is evident, surgical intervention or thermal ablation may be required to reduce the thyroid volume. After diagnosis, long-term follow-up is necessary to detect clinical hypothyroidism and follicular thyroid carcinoma early. For newborns with significant goiter, elevated TSH levels, increased FT3/FT4 ratios, elevated Tg levels, and normal anti-TPO and anti-Tg levels, genetic testing should be performed to confirm the diagnosis as it helps diagnose TPO deficiency and differentiate it from other types of CH.

In conclusion, mild TPO deficiency is an autosomal recessive disorder primarily characterized by goiter, increased FT3/FT4 ratios, elevated Tg levels, and normal anti-TPO and anti-Tg levels. Existing diagnosis is mainly based on elevated FT3/FT4 ratios and Tg levels, with genetic testing aiding confirmation. This c.1750 T > G (p.R584W) is a novel mutation in the population, expanding the mutation spectrum of mild TPO deficiency. Genetic testing of the proband and their parents may provide prenatal diagnosis for families planning to have children.

## Data Availability

The datasets presented in this study can be found in online repositories. The names of the repository/repositories and accession number(s) can be found in the article/supplementary material.
